# How can I use it? The role of functional fixedness in the survival-processing paradigm

**DOI:** 10.3758/s13423-020-01802-y

**Published:** 2020-09-15

**Authors:** Meike Kroneisen, Michael Kriechbaumer, Siri-Maria Kamp, Edgar Erdfelder

**Affiliations:** 1 School of Social Sciences, University of Mannheim, D-68131 Mannheim, Germany; 2 University of Koblenz-Landau, Landau, Germany; 3 Department of Psychology, University of Trier, Trier, Germany

**Keywords:** Memory, Evolution, Survival-processing effect, Functional fixedness

## Abstract

After imagining being stranded in the grasslands of a foreign land without any basic survival material and rating objects with respect to their relevance in this situation, participants show superior memory performance for these objects compared to a control scenario. A possible mechanism responsible for this memory advantage is the richness and distinctiveness with which information is encoded in the survival-scenario condition. When confronted with the unusual task of thinking about how an object can be used in a life-threatening context, participants will most likely consider both common and uncommon (i.e., novel) functions of this object. These ideas about potential functions may later serve as powerful retrieval cues that boost memory performance. We argue that objects differ in their potential to be used as novel, creative survival tools. Some objects may be low in functional fixedness, meaning that it is possible to use them in many different ways. Other objects, in contrast, may be high in functional fixedness, meaning that the possibilities to use them in non-standard ways is limited. We tested experimentally whether functional fixedness of objects moderates the strength of the survival-processing advantage compared to a moving control scenario. As predicted, we observed an interaction of the functional fixedness level with scenario type: The survival-processing memory advantage was more pronounced for objects low in functional fixedness compared to those high in functional fixedness. These results are in line with the richness-of-encoding explanation of the survival-processing advantage.

## Introduction

An evolutionary perspective on human memory focuses on the conditions under which our cognitive systems process information especially well (Nairne, Thompson, & Pandeirada, 2007). Consistent with ideas based on evolutionary psychology, experiments showed better memory for material that is relevant for certain adaptive ends such as social exchange (e.g., Buchner, Bell, Mehl, & Musch, 2009; Kroneisen, Woehe, & Rausch, 2015), mating (Allan, Jones, DeBruine, & Smith, 2012), or learning an association between potentially dangerous stimuli (e.g., snakes, spiders) and aversive stimuli, such as shock (see Öhman & Mineka, 2001).

In the survival-processing paradigm, participants are instructed to imagine being stranded in the grasslands of a foreign land, without any food or water, and in danger of predators. A list of items is then presented that participants are required to rate with respect to their relevance in this survival scenario. In a later surprise retention test, words encoded in this scenario are recalled better than words encoded in control scenarios such as imagining moving to a foreign country (Nairne et al., 2007) or other deep-processing tasks such as rating the pleasantness of words (e.g., Nairne, Pandeirada, & Thompson, 2008; Nairne et al., 2007).

This so-called survival-processing effect has been shown for various retention measures and different populations; it is still found when survival processing is compared to other memory-enhancing encoding tasks or alternative emotionally arousing scenarios (Kang, McDermott, & Cohen, 2008; Kroneisen & Makerud, 2017; Nairne et al., 2008; Otgaar & Smeets, 2010; Röer, Bell, & Buchner, 2013; Weinstein, Bugg, & Roediger, 2008). Furthermore, it was successfully replicated as part of the Open Science Collaboration project (Open Science Collaboration, 2015). In sum, survival processing seems to be one of the most efficient mnemonic procedures known so far (Nairne & Pandeirada, 2008a; but see Klein, 2012).

According to Nairne and colleagues, the survival-processing advantage provides evidence that human memory has been selectively tuned during evolution to process and retain information that is relevant to fitness (selective-tuning hypothesis; Nairne, Vasconcelos, & Pandeirada, 2011). This of course does not necessarily imply that a single cognitive “survival module” underlies fitness processing in general. Rather, a number of different domain-specific processes, like, for example, a predator-retention mechanism, might be involved (Nairne & Panderada, 2008b). Still, the question remains which proximate cognitive mechanisms mediate the survival-processing effect. Several explanations proposed in the literature rely on efficient forms of encoding, storage, or retrieval that are domain-general in nature and thus are associated with good memory performance in general, not just in survival contexts (see Erdfelder & Kroneisen, 2014, for a review).

One of these hypotheses, already considered by Nairne et al. (2007), posits that active elaboration and richness of encoding triggered by the rating task underlies the survival-processing advantage (Kroneisen & Erdfelder, 2011; Kroneisen, Rummel, & Erdfelder, 2014, 2016). More specifically, the richness-of-encoding hypothesis maintains that relevance ratings implicitly encourage participants to think about different uses of items in a complex survival context. These additional thoughts about potential functions generated during encoding, especially the uncommon and creative ideas, may later serve as powerful retrieval cues in a surprise memory test, thus boosting memory performance to the degree that the test is sensitive to these cues. Direct memory tests such as free recall will benefit from these processes.

Thinking about using a set of randomly compiled objects to maximize chances of survival can be considered a difficult problem-solving task (Bell, Röer, & Buchner, 2015; Kroneisen, Erdfelder, & Buchner, 2013; Röer, Bell, & Buchner, 2013). In line with this, Klein, Robertson, and Delton (2010, 2011) showed that planning (which also involves thoughts about object uses) may play a major role in the survival-processing advantage. Bell et al. (2015) even showed that instructions to think about how to use a specific item increases the memory benefit compared to the standard relevance-rating instructions. Bell and colleagues also came up with an explanation for the latter effect: When confronted with the unusual task of thinking about how an item can be used in a survival situation, participants try harder to think not only about the common functions of objects but also about novel functions. However, to produce ideas about novel functions of objects, they have to retrieve different object characteristics (e.g., form, material, stability) from long-term memory and then mentally simulate the use of the specific item for this new situation. For example, when thinking about the usefulness of bed sheets in a survival situation you can think about their normal function (e.g., cover your bed, even if the bed is just a place on the ground), as well as about novel functions (e.g., transporting things, use as towel, use as tent). In contrast, thinking about using sheets in more common contexts like moving to another city is more restricted to the usual function of the object (covering your bed). Of course, it is still possible to use a bed sheet in a more unusual way like wrap up fragile objects. However, there is often no need to use items outside their normal function in a moving scenario. In line with this, Bell et al. (2015) reanalyzed data from Röer et al. (2013) and found that ideas produced under survival instructions received higher creativity ratings from raters than the ideas generated in the moving condition. Furthermore, Wilson (2016) showed that the survival scenario elicited more alternative uses in the Guilford’s Alternate Uses Test compared to non-survival-related conditions.

However, the possibility of using objects in ways that differ from their common function vary considerably between objects. Some items can be used in many novel ways while others are more or less restricted to their typical everyday function. We refer to such differences between objects as differences in *functional fixedness*. The term “functional fixedness” was already used by Duncker (1945), and is an important phenomenon in problem-solving research. In essence, it means that people focus on a specific (common) function of an object while overlooking other possible functions that might help to solve a problem (Arnon & Kreitler, 1984). For example, in the candle problem (Duncker, 1945), participants are instructed to fix and light a candle on a wall. However, they only have a matchbox and a box of thumbtacks besides the candle to accomplish this task. To solve this problem, participants have to empty the box of thumbtacks, use the thumbtacks to nail the box to the wall, put the candle into the box, and light the candle with the match. Functional fixedness means that participants struggle to see the box as a device to hold the candle.

In line with these ideas, a string, for example, can be seen as an object with many possibilities of using it in novel ways. The functional fixedness of the object is thus low. In contrast, a traffic light, for example, is more restricted to its common function. Consequently, its functional fixedness is high. This functional fixedness is independent from the context in which these objects occur. In the following experiment, we aimed to test whether functional fixedness of objects affects the strength of the survival-processing advantage as predicted – that is, low functional fixedness is associated with stronger memory benefits than high functional fixedness of objects.

## Pre-studies

As explained above, the richness-of-encoding hypothesis maintains that the survival-processing advantage is due to the stronger stimulation of ideas about the possible uses of objects in survival compared to control contexts. These unique ideas may later act as highly distinctive retrieval cues in the retention task (Kroneisen & Erdfelder, 2011).

Bell et al. (2015) suggested that the survival condition triggers participants to think about not only common but also novel functions of objects. However, items differ in their potential to use them in novel ways. The more novel functions someone can think of, the more unique retrieval cues should be available to guide the person in the later memory test.

## Pre-study 1

Based on this reasoning, we wanted to create a list of words that differ in their level of functional fixedness.

### Method

#### Participants

Thirteen participants from the University of Koblenz-Landau were asked to rate 32 words for concreteness, meaningfulness, and vividness.

#### Design and procedure

In order to find a list of words, one of the authors searched through the German dictionary to obtain a list of items that can be assigned to either (a) items very restricted to their common function such as a desktop (high functional fixedness) or (b) items that can be used in many different and novel ways such as a bed sheet (low functional fixedness). This resulted in a list of 141 concrete words. In the next step, 15 participants rated the degree of functional fixedness of these 141 concrete words on a scale ranging from -2 (item can only be used for one specific thing) to +2 (item can be used for a range of different things). A total of 16 concrete words highest in functional fixedness (*M*_highFF_ = -1.23, *SD*_highFF_ = 0.76) and 16 concrete words lowest in functional fixedness (*M*_lowFF_ = 1.23, *SD*_lowFF_ = 0.27) were chosen (see the Appendix for the full lists of words). Thus, overall 32 target words were chosen. In a final step, different participants were asked to rate these 32 words for concreteness, meaningfulness, and vividness on a scale ranging from 1 (abstract; low in meaningfulness; low in vividness) to 5 (concrete; high in meaningfulness; high in vividness).

### Results

Differences between words high and low in functional fixedness were negligible on all three dimensions (Table 1).

**Table 1:** Means and standard deviations of participants’ ratings for concreteness, meaningfulness, and vividness, shown separately for words high and low in functional fixedness

	High functional fixedness	Low functional fixedness
	*Mean (SD)*	*Mean (SD)*
Concreteness	4.28 (0.53)	4.24 (0.46)
Meaningfulness	3.29 (0.88)	2.97 (0.97)
Vividness	3.96 (0.65)	4.10 (0.57)

## Pre-study 2

As mentioned above, we argue that objects high and low in functional fixedness differ in their potential to use them in novel ways. In order to test this for the words selected in Pre-study 1, we conducted another pre-study.

### Method

#### Participants

Thirty-seven psychology students (29 females) from the University of Mannheim and the University of Trier participated for course credits. None of these participants took part in Pre-study 1. Their age ranged from 19 to 40 years (*M* = 23.16, *SD* = 5.86). Given *N* = 37, *α* = .05, and *df* = 36, a one-tailed matched-pairs *t-*test can detect a medium effect size *d* = 0.5 (Cohen, 1988) with a power of 1-β = .91 (Faul, Erdfelder, Lang, & Buchner, 2009).

#### Design and procedure

Participants were tested online in sessions that lasted approximately 20 min. After providing informed consent, participants responded to several items assessing demographic information. Next, participants were told that they will see the names of 32 objects for 30 s each. We asked them to provide us with different ideas of how to use these objects. We explicitly told our participants that this task may be easy for some objects and harder for others and instructed them to think of as many ideas as possible for each object. Each word was shown for exactly 30 s, immediately followed by the next word. This task was preceded by a short practice trial, in which the participants had to provide ideas for how to use the object “blanket” for 30 s. Then, the 32 words from Pre-study 1 were shown to each participant in random order.

#### Results

We counted the number of ideas each participant provided. For words low in functional fixedness more ideas were created (*M*_lowFF_ = 3.89, *SD*_lowFF_ = 1.19) than for words high in functional fixedness (*M*_highFF_ = 2.97, *SD*_highFF_ = 1.27; *t*(36) =  − 9.58, *p* < .001, estimated d =  − 0.75). In line with the ratings provided in Pre-study 1, words low in functional fixedness enabled participants to generate more ideas about potential uses than words high in functional fixedness.

## Main experiment

Using the word sets preselected and validated in Pre-studies 1 and 2, we tested our hypothesis that functional fixedness of objects affects the number of words recalled. Specifically, words describing objects low in functional fixedness should be recalled better. Furthermore, we predicted an interaction between functional fixedness and scenario: Words low in functional fixedness should benefit more from the survival-processing advantage than words high in functional fixedness.

### Method

#### Participants

One hundred and forty-one students (125 female) from the University of Koblenz-Landau participated. None of these participants took part in the pre-studies. They received a monetary compensation. Their age ranged from 18 to 31 years (*M* = 21.59, *SD* = 2.37). For a medium effect size *f* = 0.25 (Cohen, 1988), *α* = .05, and *df*_1_ = 1, *df*_2_ = 139, the power to detect a significant interaction of a between-Ss and a within-Ss factor in our design (see below) exceeds .99 (Faul et al., 2009).

#### Apparatus and materials

We used the 32 words from the pre-studies as targets in the encoding phase, of which 16 were high and 16 were low in functional fixedness. To absorb primacy and recency effects, we added six buffer words, three at the beginning and three at the end of the list. Apart from the buffer words, all words were presented in random order. Except for language, the survival and moving scenario descriptions were identical to those used by Nairne et al. (2007). All materials were presented in German.

#### Design

A 2 (scenario: survival vs. moving) × 2 (functional fixedness: high vs. low) mixed design was used. Participants were randomly assigned to either the survival (*N* = 71) or the moving (*N* = 70) scenario (between-subjects factor). Participants of both conditions rated 32 words according to their relevance. These words were either high (16 items) or low (16 items) in functional fixedness (within-subjects factor).

Recall performance, response latencies, and relevance ratings served as dependent variables.

#### Procedure

Participants were tested in groups ranging in size from one to four in a lab in Landau. Each session lasted approximately 25 min. Stimuli were presented and controlled by personal computers, and participants entered their responses using the keyboard.

Depending on the experimental condition, participants were asked to rate words according to their relevance for either the survival or the moving scenario. Stimuli were presented one at a time for 5 s each, and participants were asked to rate the words on a 5-point scale, with 1 indicating “absolutely not relevant” and 5 “extremely relevant” to the current scenario. They had to respond within 5 s. If they did not respond within this time limit, a warning message occurred and the next word was presented. This would result in a trial without rating or response-time acquisition. The rating task was preceded by a short practice trial, in which two words had to be rated for relevance. After the rating task, participants performed a distractor task (i.e., filling in an unrelated questionnaire) for 12 min and were then unexpectedly prompted with a free-recall test for the words previously processed in the relevance-rating task. Similar to other experiments using the survival-processing paradigm (e.g., Kroneisen & Makerud, 2017), the final recall phase lasted for 8 min. It was not possible to terminate the recall phase earlier than 8 min. At the end of the experiment, participants were debriefed and thanked for their participation.

### Results

The significance level was set to α = .05 for all statistical tests. Relevance ratings were provided for 99.93% of the presented words. Overall, there were 32 missed trials. Eighteen subjects did not provide a response for all the words presented to them.

**Fig. 1:**
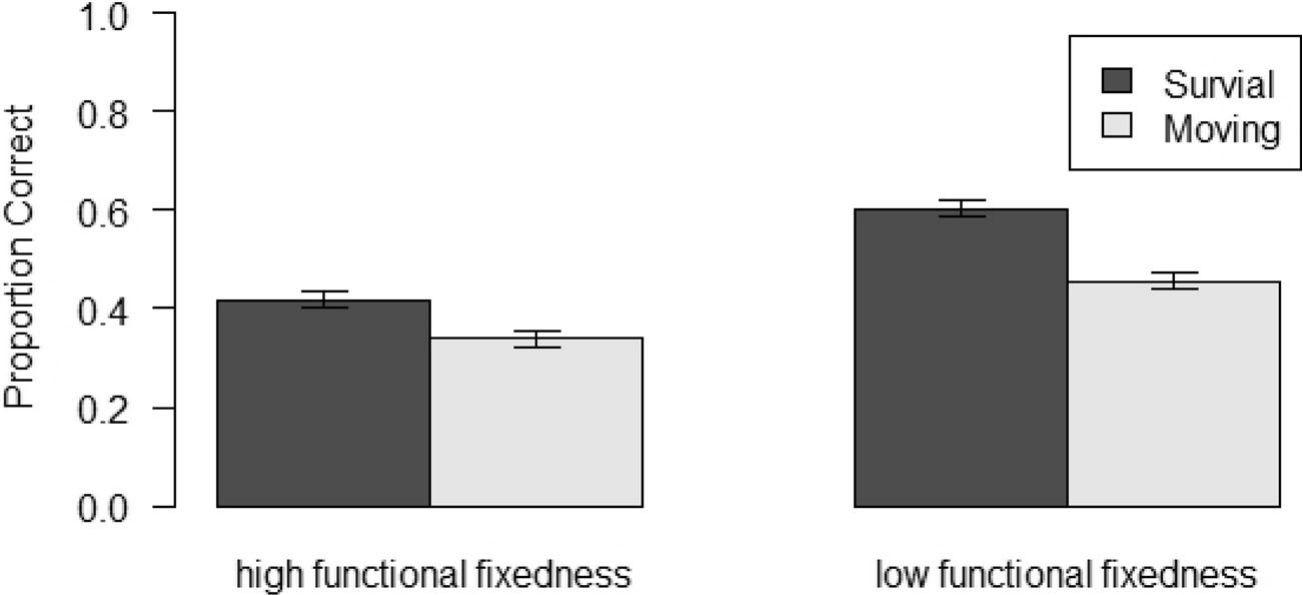
Mean proportion of correct recall for each scenario, shown separately for words high and low in functional fixedness. The error bars represent standard errors of the means

The mean proportions of correct free recall for both scenarios, separately for words high and low in functional fixedness, are shown in Fig. 1 (*M*_SurvivalFFhigh_ = .42, *M*_SurvivalFFlow_ = .60, *M*_MovingFFhigh_ = .34, *M*_MovingFFlow_ = .46; range of words recalled: 5–25). A 2 (scenario) × 2 (functional fixedness) mixed ANOVA revealed a significant main effect of scenario, $F\left(1,139\right)=31.70,p<.001,{\upeta}_p^2\kern0.5em =.19$. Words processed in a survival scenario were recalled better than words processed in a moving scenario. There was also a significant main effect of functional fixedness, $F\left(1,139\right)=135.50,p<.001,{\upeta}_p^2\kern0.5em =.49$. Words low in functional fixedness were remembered better than words high in functional fixedness. The interaction between scenario and functional fixedness was also significant, $F\left(1,139\right)=8.32,p=.005,{\upeta}_p^2\kern0.5em =.06.$

Table 2 presents the median response times for the relevance ratings, separately for each scenario and word type. Response times for the ratings did not differ significantly between the scenarios, $F\left(1,139\right)=1.58,p=.21,{\upeta}_p^2\kern0.5em =.01.$ There was a main effect for functional fixedness, $F\left(1,139\right)=14.65,p<.001,{\upeta}_p^2\kern0.5em =.10.$Response times for the words high in functional fixedness were significantly longer than those for words low in functional fixedness. There was also a significant interaction between scenario and functional fixedness, $F\left(1,139\right)=27.43,p<.001,{\upeta}_p^2\kern0.5em =.16$, indicating that response times for ratings took longest when words were high in functional fixedness and processed in the survival scenario.

**Table 2:** Means and standard errors of participants’ median rating latencies for each scenario, shown separately for words high and low in functional fixedness

Scenario	Fixedness	Rating latency (ms)
		*Mean* (SEM)
Survival	High	2,373.44 (56.39)
	Low	2,052.49 (56.32)
Moving	High	2,098.33 (56.39)
	Low	2,148.24 (56.32)

**Fig. 2:**
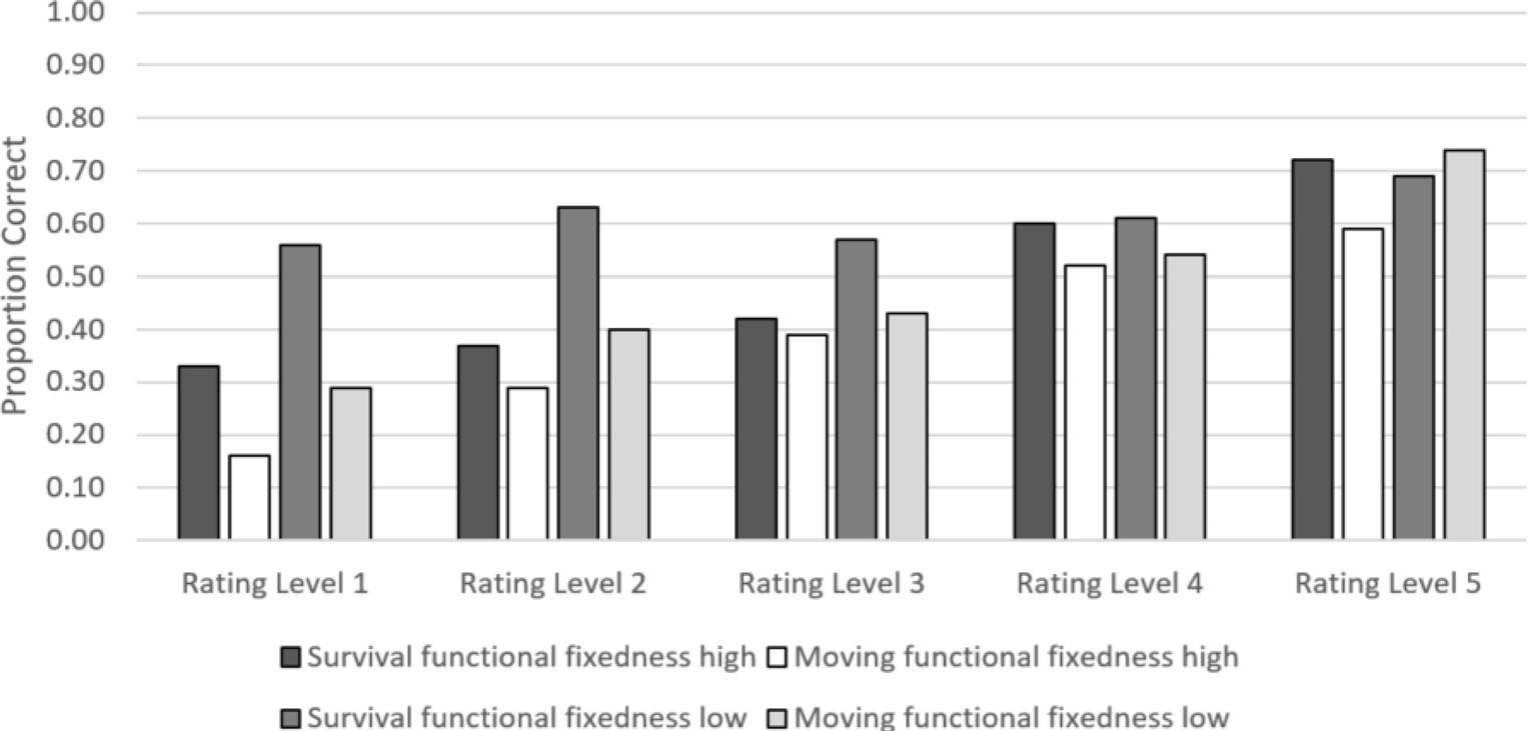
Mean proportions of correct recall for each scenario, shown separately for words high and low in functional fixedness and each rating category. The error bars represent standard errors of the means

Figure 2 displays recall performance as a function of the relevance ratings provided in the encoding phase. Obviously, higher relevance ratings are associated with higher levels of recall. Controlling for overall recall performance of the participants, in the survival condition the partial correlation between ratings and recall rates was significant for the words high (*r* = .24; *p* < .001) and low (*r* = .08; *p* < .009) in functional fixedness. The same pattern can be found for words processed in the moving scenario (partial correlation for words high in functional fixedness: *r* = .32; *p* < .001, and words low in functional fixedness: *r* = .23; *p* < .001). We also tested whether and – if so how – the effects of scenario and functional fixedness on recall performance are moderated by the relevance ratings the words receive in the encoding phase. To answer this question, it was necessary to combine ratings to cruder rating categories. Working with the original ratings would have implied a loss of 107 participants from the analysis because not every participant made use of every possible rating category. To avoid such a serious loss of data, we split the ratings in two categories with low (i.e., ratings 1–3) versus high (i.e., ratings 4 and 5) relevance judgments. Using this approach, only 13 participants had to be excluded because they did not provide data for every rating category (low vs. high), leaving a sample of *N* = 128 participants for analysis. Table 3 presents the mean recall proportions of this sample for the low (left side) versus high (right side) rating categories, separately for scenario and functional fixedness. A 2 (scenario) × 2 (functional fixedness) × 2 (rating categories) mixed ANOVA revealed significant main effects of scenario ($F\left(\mathrm{1,126}\right)=15.40,p<.001,{\upeta}_p^2\kern0.5em =.11$), functional fixedness ($F\left(\mathrm{1,126}\right)=48.04,p<.001,{\upeta}_p^2\kern0.5em =.28$), and ratings, $F\left(\mathrm{1,126}\right)=126.73,p<.001,{\upeta}_p^2\kern0.5em =.50$. The interactions between scenario and ratings ($F\left(\mathrm{1,126}\right)=7.83,p=.006,{\upeta}_p^2\kern0.5em =.06\Big),$and between functional fixedness and ratings was also significant, $F\left(\mathrm{1,126}\right)=26.72,p<.001,{\upeta}_p^2\kern0.5em =.17$. The interaction between scenario and functional fixedness was not significant, $F\left(\mathrm{1,126}\right)<0.01,p=.99,{\upeta}_p^2\kern0.5em <.001$. More importantly, the three-way interaction between scenario, functional fixedness, and ratings was significant, ($F\left(\mathrm{1,126}\right)=17.04,p<.001,{\upeta}_p^2\kern0.5em =.12$), showing that the two-way interaction between scenario and functional fixedness evident in the aggregate data (see Fig. 1) is very pronounced for words with low relevance ratings and does not show up in high ratings (see Table 3). Also, the main effects of scenario and functional fixedness largely disappear for words with high relevance ratings, as evidenced by the significant two-way interactions involving the rating level.

**Table 3:** Means and standard deviation of participants’ recall proportions for each scenario, shown separately for words high and low in functional fixedness and words receiving low (1–3) versus high (4–5) relevance ratings

	Low rating (1–3)		High rating (4–5)	
	Functional fixedness		Functional fixedness	
	High	Low	High	Low
	*Mean (SD)*	*Mean (SD)*	*Mean (SD)*	*Mean (SD)*
Survival	0.34 (0.03)	0.60 (0.03)	0.66 (0.03)	0.63 (0.03)
Moving	0.25 (0.03)	0.38 (0.03)	0.55 (0.03)	0.65 (0.03)

### Discussion

It is reasonable to assume that our memory systems have evolved to help us survive and thereby enhance our fitness (Nairne & Pandeirada, 2010). Researchers suggested that the survival-processing effect reflects how specific selection pressures shaped these memory systems (Nairne & Pandeirada, 2008b, 2010; Weinstein et al., 2008). In line with these ideas, the survival-processing advantage proved to be a very robust and stable effect that is found in different populations (e.g., Kroneisen & Makerud, 2017; Nairne et al., 2007; Otgaar et al., 2010). It was replicated both in older adults (Nouchi, 2012) and in children (Aslan & Bäuml, 2012; Otgaar & Smeets, 2010). These well-replicated results have been argued to guide our understanding of cognitive “biases or tunings” (Nairne & Pandeirada, 2010, p.2) that helped humans survive in ancestral environments.

However, our understanding of the survival-processing effect would remain incomplete without an understanding of the proximate cognitive mechanisms producing this benefit. One class of proximate explanations focuses on the nature of incidental encoding during the relevance-rating task. The richness of encoding hypothesis maintains that survival processing stimulates participants to generate a variety of both usual and unusual ideas on how to use objects to survive (e.g., using a chair to fight off a tiger). These ideas may later serve as powerful retrieval cues in the unexpected memory test. In contrast, scenarios such as the moving scenario provide less opportunity to come up with divergent ideas about how to use these objects (e.g., a chair can be used to sit on it or to stand on it while attaching something). Consistent with this hypothesis, Röer et al. (2013) showed that the survival scenario encourages participants to generate more ideas about how to use items. Furthermore, the strength of the survival-processing advantage tends to increase with the number of unique relevance arguments generated per item. Consequently, treatments that hinder or interfere with the generation of many creative ideas in the survival scenario reduce or even abolish the effect (Kroneisen & Erdfelder, 2011; Kroneisen et al., 2013; Kroneisen et al., 2014, 2016).

Bell et al. (2015) argued that thinking about novel uses of objects requires retrieving characteristics of these objects (e.g., form, material, stability) from long-term memory and then simulating their use in the given situation. We showed that objects differ in their potential to use them in novel ways. Some items can be used in many different ways. These items have a very low level of functional fixedness. Other items can mainly be used for their common function. They have a high degree of functional fixedness. Based on these ideas, we created wordlists of objects that differed in their level of functional fixedness. We assumed that objects low in functional fixedness allow participants to create many different and novel using functions, independently of the scenario. Therefore, for these words, a higher number of retrieval cues can be created and used in the later recall task. This effect should be especially pronounced in the survival condition because novel, creative ideas that maximize chances of survival are a key component of this scenario. The moving scenario, in contrast, prompts people to think about prototypical uses of objects in the first place.

We experimentally tested the effect of an item’s functional fixedness on memory performance in the survival and the moving scenario. Replicating prior findings, participants who evaluated words in the context of an imagined survival scenario demonstrated enhanced performance on a later memory test. In addition, we observed an effect of functional fixedness: Words low in functional fixedness were remembered better. Furthermore, we found a significant interaction between functional fixedness and scenario: The survival-processing benefit was strongest for words low in functional fixedness. All three effects are in line with our hypotheses.

Participants took more time to rate the relevance of words high in functional fixedness. Furthermore, we found that ratings required most time when the words high in functional fixedness were processed in the survival scenario, suggesting that participants struggled to find useful functions for these objects, especially in the survival condition.

Consistent with previous findings (e.g., Aslan & Bäuml, 2012; Butler et al., 2009; Kroneisen & Makerud, 2017; Nairne et al., 2007), congruity had an effect on memory performance: Higher relevance ratings were associated with higher recall rates. Overall, items that are congruent with or relevant for the processing task are remembered better. One interpretation of these congruency effects is that congruent items stimulate more ideas about possible uses of these objects (Bell, Röer, & Buchner, 2013). Röer et al. (2013) demonstrated that congruency effects in the survival-processing paradigm can be explained by the number of ideas generated during encoding. Their participants generated more ideas in response to scenario-congruent than to scenario-incongruent items.

In our experiment, both the survival-processing advantage and the low-functional fixedness advantage hold even if the relevance-rating level is controlled for (see Fig. 2). This finding is important because it shows that both the survival-processing advantage and the low-functional fixedness benefit cannot be explained as by-products of simple congruency effects. Moreover, either effect tends to be more pronounced for low ratings compared to high ratings (cf. Fig. 2). Why should this be the case? As discussed above, the survival scenario encourages participants to generate creative ideas about how to use items. This effect should be strongest for items that do not fit in the scenario (i.e., objects with low relevance ratings), because in these cases people are forced to think about novel uses of these objects. In addition, it is easier to find novel uses for items with low functional fixedness, resulting in strongest memory benefits when survival-processing refers to scenario-incongruent items low in functional fixedness. Thus, the observed result pattern is perfectly in line with the richness of the encoding explanation of survival-processing benefits.

In sum, we learned more about the processes underlying the survival-processing effect. Our results support the idea that survival processing involves thinking about common and novel functions of objects when rating the relevance of items. In contrast, the moving scenario mainly involves thinking about common uses of objects. The more novel and distinct functions of objects a person is able to generate, the better later memory retrieval. As shown in our present research, both the stimulating survival-processing context and low functional fixedness of objects contribute to this memory advantage.

#### Open practices statement

The word material for the main experiment can be found in the Appendix. The experiment was not preregistered.

## Appendix

**Table 4:** Word material used in the experiment. In the English translation the same word (battery) is used for two different German words (Akku and Batterie)

German word	English word	Functional fixedness (high vs. low)	Mean functional fixedness rating (-2/+2)
Akku	Rechargeable battery	High	-1.33
Ampel	Traffic light	High	-1.80
Anker	Anchor	High	-1.13
Batterie	Battery	High	-1.47
Bügeleisen	Flat iron	High	-1.53
Desktop	Desktop	High	-1.53
Gießkanne	Watering can	High	-1.27
Lampe	Lamp	High	-1.27
Pfeife	Pipe (smoking)	High	-1.27
Radio	Radio	High	-1.80
Reißverschluss	Zipper	High	-1.13
Tacker	Stapler	High	-1.60
Taschenlampe	Flashlight	High	-1.27
Waage	Weighing scale	High	-1.87
Walze	Roller	High	-1.07
Winkelmaß	Goniometer	High	-1.33
Alufolie	Tinfoil	Low	1.27
Band	Ribbon	Low	1.13
Brett	Plank	Low	1.47
Draht	Wire	Low	1.27
Eimer	Bucket	Low	1.07
Faden	Thread	Low	1.33
Holz	Timber	Low	1.87
Karton	Carton	Low	1.13
Laken	Sheet	Low	0.80
Netz	Net	Low	1.27
Palette	Palette	Low	1.07
Papier	Paper	Low	1.73
Sack	Sack	Low	1.07
Schnur	String	Low	1.13
Stein	Stone	Low	1.07
Stock	Stick	Low	1.07

## References

[CR1] Allan K, Jones BC, DeBruine LM, Smith DS (2012). Evidence of adaptation for mate choice within women’s memory. Evolution and Human Behavior.

[CR2] Arnon R, Kreitler S (1984). Effects of meaning training on overcoming functional fixedness. Current Psychological Research & Reviews.

[CR3] Aslan A, Bäuml K-H (2012). Adaptive memory: Young children show enhanced retention of fitness-related information. Cognition.

[CR4] Bell R, Röer JP, Buchner A (2013). Adaptive memory: The survival-processing advantage is not due to negativity or mortality salience. Memory & Cognition.

[CR5] Bell R, Röer JP, Buchner A (2015). Adaptive memory: Thinking about function. Journal of Experimental Psychology: Learning, Memory, and Cognition.

[CR6] Buchner A, Bell R, Mehl B, Musch J (2009). No enhanced recognition memory, but better source memory for faces of cheaters. Evolution and Human Behavior.

[CR7] Butler AC, Kang SHK, Roediger HL (2009). Congruity effects between materials and processing tasks in the survival-processing paradigm. Journal of Experimental Psychology: Learning, Memory, and Cognition.

[CR8] Cohen J (1988). *Statistical power analysis for the behavioral sciences*.

[CR9] Duncker K (1945). *On problem-solving* (L. S. Lees, Trans.). Psychological Monographs.

[CR10] Erdfelder E, Kroneisen M, Schwartz BL, Howe M, Toglia M, Otgaar H (2014). Proximate cognitive mechanisms underlying the survival-processing effect. *What is adaptive about adaptive memory?*.

[CR11] Faul F, Erdfelder E, Buchner A, Lang AG (2009). Statistical power analyses using G*Power 3.1: Tests for correlation and regression analyses. Behavior Research Methods.

[CR12] Kang SHK, McDermott KB, Cohen SM (2008). The mnemonic advantage of processing fitness-relevant information. Memory & Cognition.

[CR13] Klein SB (2012). A role for self-referential processing in tasks requiring participants to imagine survival on the savannah. Journal of Experimental Psychology: Learning, Memory, and Cognition.

[CR14] Klein SB, Robertson TE, Delton AW (2010). Facing the future: Memory as an evolved system for planning future acts. Memory & Cognition.

[CR15] Klein SB, Robertson TE, Delton AW (2011). The future orientation of memory: Planning as a key component mediating the high levels of recall found with survival-processing. Memory.

[CR16] Kroneisen M, Erdfelder E (2011). On the plasticity of the survival-processing effect. Journal of Experimental Psychology: Learning, Memory, and Cognition.

[CR17] Kroneisen M, Erdfelder E, Buchner A (2013). The proximate memory mechanism underlying the survival-processing effect: Richness of encoding or interactive imagery?. Memory.

[CR18] Kroneisen M, Makerud E (2017). The effects of item material on encoding strategies: Survival processing compared to the Method of Loci. Quarterly Journal of Experimental Psychology.

[CR19] Kroneisen M, Rummel J, Erdfelder E (2014). Working memory load eliminates the survival-processing effect. Memory.

[CR20] Kroneisen M, Rummel J, Erdfelder E (2016). What kind of processing is survival-processing? Effects of different types of dual-task load on the survival-processing effect. Memory & Cognition.

[CR21] Kroneisen M, Woehe L, Rausch LS (2015). Expectancy effects in source memory: How moving to a bad neighborhood can change your memory. Psychonomic Bulletin & Review.

[CR22] Nairne JS, Pandeirada JNS (2008). Adaptive memory: Is survival-processing special?. Journal of Memory and Language.

[CR23] Nairne JS, Pandeirada JNS (2008). Adaptive memory: Remembering with a stone-age brain. Current Directions in Psychological Sciences.

[CR24] Nairne JS, Pandeirada JNS (2010). Adaptive memory: ancestral priorities and the mnemonic value of survival-processing. Cognitive Psychology.

[CR25] Nairne JS, Pandeirada JNS, Thompson S (2008). Adaptive memory: the comparative value of survival-processing. Psychological Science.

[CR26] Nairne JS, Thompson SR, Pandeirada JNS (2007). Adaptive memory: survival-processing enhances retention. Journal of Experimental Psychology: Learning, Memory, and Cognition.

[CR27] Nairne JS, Vasconcelos M, Pandeirada JNS, Seel NM (2011). Adaptive memory and learning. *Encyclopedia of the sciences of learning*.

[CR28] Nouchi R (2012). The effect of aging on the memory enhancement of the survival judgment task. Japanese Psychological Research.

[CR29] Öhman A, Mineka S (2001). Fears, phobia, and preparedness: Toward an evolved module of fear and fear learning. Psychological Review.

[CR30] Otgaar H, Smeets T (2010). Adaptive memory: Survival processing increases both true and false memory in adults and children. Journal of Experimental Psychology: Learning, Memory, and Cognition.

[CR31] Otgaar, H., Smeets, T., & van Bergen, S. (2010). Picturing survival memories: Enhanced memory after fitness-relevant processing occurs for verbal and visual stimuli. *Memory & Cognition*, 38, 23-28. 10.3758/MC.38.1.23 10.3758/MC.38.1.23 19966235

[CR32] Röer JP, Bell R, Buchner A (2013). Is the survival-processing memory advantage due to richness of encoding?. Journal of Experimental Psychology: Learning, Memory, and Cognition.

[CR33] Weinstein Y, Bugg JM, Roediger HL (2008). Can the survival recall advantage be explained by the basic memory processes?. Memory and Cognition.

[CR34] Wilson S (2016). Divergent thinking in the grasslands: Thinking about object function in the context of a grassland survival scenario elicits more alternate uses than control scenarios. Journal of Cognitive Psychology.

